# Meckel's diverticulum causing acute intestinal obstruction: A case report and comprehensive review of the literature

**DOI:** 10.1016/j.amsu.2022.103734

**Published:** 2022-05-07

**Authors:** Talal Almas, Abdulla K. Alsubai, Danyal Ahmed, Muneeb Ullah, Muhammad Faisal Murad, Khadeer Abdulkarim, Eissa Sultan Alwheibi, Mohamed Alansaari, Tala Abdullatif, Sebastian Hadeed, Muhammad Omer Khan, Majid Alsufyani, Enaam Alzadjali, Arjun Samy, Mert Oruk, Mhmod Kadom, Fatemah Saleh Alhajri, Ahmed Barakat, Maen Monketh Alrawashdeh, Mohammad Said, Reem AlDhaheri, Emad Mansoor

**Affiliations:** a Royal College of Surgeons in Ireland, Dublin, Ireland; b Maroof International Hospital, Islamabad, Pakistan; c National University of Ireland Galway, Galway, Ireland; d Division of Gastroenterology and Liver Disease, University Hospitals Cleveland Medical Center, Case Western Reserve University, Cleveland, OH, USA

**Keywords:** Meckel's diverticulum, Acute intestinal obstruction

## Abstract

**Introduction:**

Meckel's diverticulum is a congenital anomaly that is often detected incidentally. When it presents symptomatically, it causes painless gastrointestinal bleeding. Nevertheless, in rare instances, it can cause acute intestinal obstruction, often obscuring the true clinical picture.

**Case presentation:**

A 31-year-old male presented to the emergency department with a 24-h history of unremitting nausea, biliary emesis, abdominal distension, and absolute constipation. After ruling out the most common etiologies of acute bowel obstruction, radiological imaging was obtained and was suggestive of meckel's diverticulum. Laparoscopic meckel's diverticulectomy was performed, with the subsequent histopathological analysis confirming ectopic gastric tissue.

**Discussion:**

Meckel's diverticulum occurs consequent to incomplete obliteration of the vitelline or omphalomesenteric duct, which connects the developing intestines to the yolk sac. It is found in roughly 2% of the population, of which only about 4% may become symptomatic due to any number of complications. Specifically, small bowel obstruction (SBO) and diverticulitis secondary to ectopic gastric or pancreatic tissue are the most common presentations of symptomatic MD.

**Conclusion:**

Although relatively rare in adults, MD should be considered in the list of differentials in patients with intussusception leading to SBO, especially on a background history unremarkable for the most common etiologies causing SBO including post-operative adhesions and hernias.

## Highlights

• Meckel's diverticulum, a rare congenital abnormality, usually presents with painless bleeding. However, in rare instances, it might cause acute intestinal obstruction, often obscuring the clinical picture.
• In patients presenting with acute intestinal obstruction on a background history unremarkable for the most common etiologies, meckel's diverticulitis should be considered in the list of differentials.
• In such instances, meckel's diverticulectomy remains the mainstay of surgical management, yielding a prompt resolution of obstructive symptoms.

## 1 Introduction

Acute intestinal obstruction continues to be a medical and surgical emergency that warrants an emergency intervention. Acute bowel obstruction usually presents with a vague constellation of symptoms, often characterized by bilious/non-bilious emesis, nausea, vomiting, anorexia, and abdominal pain [1]. While postoperative adhesions and tumors afflicting the bowel remain leading causes of acute intestinal obstruction, rarer entities, such as Meckel's diverticulum, can seldom be the source, often obscuring the true clinical picture [2,3]. In gastroenterology literature, post-operative adhesions and hernias are frequently cited as being the leading causes of small bowel obstruction. However, rare congenital abnormalities such as meckel's diverticulum are not routinely thought to be the source of acute small bowel obstruction [2,3]. Meckel's diverticulum (MD) is defined as a congenital anomaly that ensues in the wake of partial closure and persistence of the vitelline, or the omphalomesenteric, duct during embryogenesis [1,2]. This usually occurs in the fifth week of development and causes a true outpouching of the small intestine, located approximately two feet from the ileocecal valve [2,3]. It is the most common congenital abnormality afflicting the gastrointestinal tract and has been reported in up to 1–3% of patients [2]. MD, a true diverticulum, involves all layers of the small intestine and is known to contain ectopic gastric mucosa [1]. It is generally asymptomatic and is usually discovered incidentally during surgical exploration of other diseases or less commonly through diagnostic imaging [1]. However, hemorrhagic, inflammatory, and obstructive complications can arise [[1], [2], [3]]. Additionally, MD can present with painless bleeding due to ectopic gastric acid and pepsin production in the diverticulum (gastric mucosa or pancreatic differentiation in MD mucosa), further complicating the clinical picture [3,4]. In patients presenting with painless gastrointestinal bleeding of unknown etiology, MD may be suspected [2,3]. Nonetheless, acute intestinal obstruction consequent to the presence of meckel's diverticulum in the adult population remains a clinical enigma [3,4]. Herein, we elucidate the case of a 31-year-old male who presented with chief complaints of nausea, biliary emesis, and abdominal distension on a background of unremarkable medical and surgical history. Further investigative workup divulged the presence of meckel's diverticulum, with subsequent diverticulectomy resulting in prompt abatement of the patient's symptoms. The overarching objective of the present paper is to prompt clinicians to recognize MD as a potential cause of acute small bowel obstruction. Although the patient's age at presentation can yield imperative diagnostic cues, MD should nevertheless be considered in the list of differentials in patients presenting with acute small bowel obstruction regardless of age.

## 2 Case presentation

A 31-year-old male presented to the emergency department with a 24-h history of unremitting nausea, biliary emesis, abdominal distension, and absolute constipation. Notably, the patient's last regular bowel movement had been three days prior to the current presentation, with the patient erroneously attributing his altered bowel habits to his recent onset of anorexia of unknown origin. The patient reported no red flag symptoms, affirmatively denying recent fevers, previously altered bowel habits, infectious urinary symptoms, or weight loss. The patient's prior medical and surgical histories were unimpressive, and he reported no other comorbidities. Pertinently, the patient had had an episode similar to the current one four years ago; at the time, his condition was managed conservatively and resulted in a prompt resolution of his symptoms within 24 hours of his presentation to the hospital. The patient remained asymptomatic thereafter with no consequent episodes till the current presentation.

**Fig. 1:**
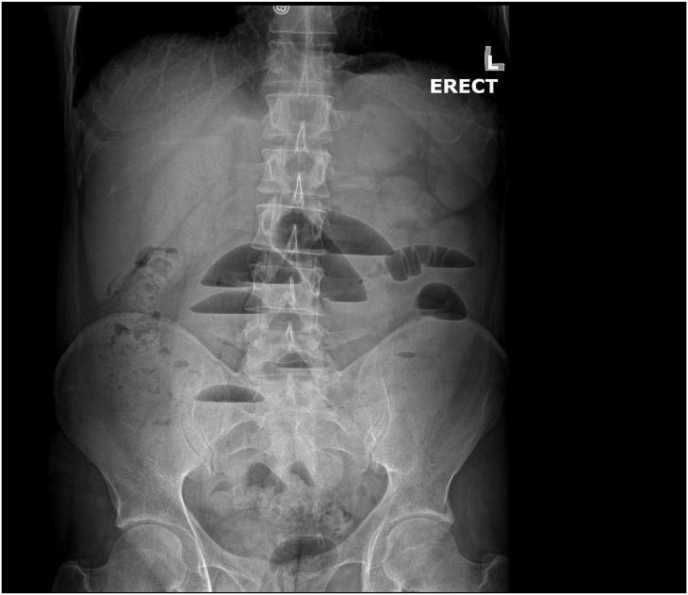
An abdominal x-ray divulging multiple air-fluid levels within the colon, thus alluding to an obstructive etiology.

Upon clinical examination, the patient appeared profusely unwell, with excruciating, 7/10, vague, non-localized abdominal pain that caused significant distress to the patient. Abdominal examination revealed a soft, distended abdomen that was non-tender with no evidence of rigidity, peritonitis, or guarding. Pertinently, bowel sounds were audible and were noted to be tinkling in nature, raising the initial suspicions for an obstructive etiology underlying the patient's clinical presentation. The murphy's sign, along with the psoas, obturator, and rovsing's signs, were all negative and unimpressive, effectively ruling out acute cholecystitis and acute appendicitis as plausible etiologies. The patient's past surgical history was also unimpressive, further precluding post-operative adhesions as the likely etiology. Upon clinical examination, there was no evidence of hernial protrusions, and the genital orifices were unremarkable. Furthermore, clinical examination of the abdomen did not reveal any rigidity or guarding, and no signs of peritonitis were appreciated. The patient's C-reactive protein was raised to 15; however, his remaining labs were unremarkable for any pertinent derangements. In order to better delineate the etiology underlying the patient's presentation, an abdominal x-ray was obtained and revealed multiple air-fluid levels in the small bowel (Fig. 1).

**Fig. 2:**
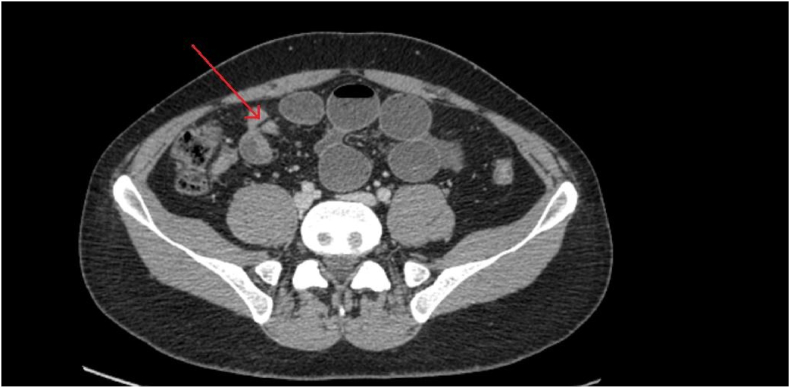
CT scan of the patient's abdomen showing a transition point in the terminal ileum (red arrow), with mesenteric band cut-off. (For interpretation of the references to colour in this figure legend, the reader is referred to the Web version of this article.)

Based on the patient's x-ray findings, an obstructive pathology was deemed plausible. However, given that the patient demonstrated an unremarkable surgical history and did not show signs of a possible gastrointestinal malignancy, adhesions, and tumor as causes of acute intestinal obstruction were effectively ruled out. Further radiological investigation through the means of a computed tomography (CT) scan divulged a transition point in the terminal ileum, with mesenteric band cut-off, strongly alluding to the presence of a diverticulum in the specified region (Fig. 2).

**Fig. 3:**
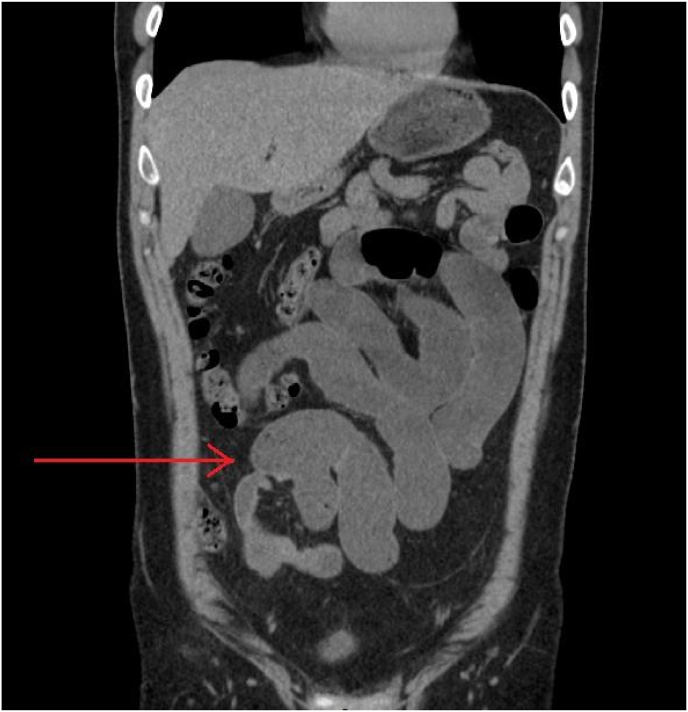
CT scan of the abdomen showing the presence of meckel's loops (red arrow), further alluding to the presence of meckel's diverticulum. (For interpretation of the references to colour in this figure legend, the reader is referred to the Web version of this article.)

The CT imaging of the abdomen further revealed the presence of the classical meckel's loops, thereby confirming the presence of meckel's diverticulum (Fig. 3).

Considering the impression obtained from the patient's radiological work-up, a multidisciplinary team meeting (MDT) was conducted. The presence of meckel's diverticulum due to an acute small bowel obstruction was deemed exceedingly plausible. Consequently, the patient was managed using conservative measures for the next 24 hours; however, the patient refused a nasogastric tube, which would have been pivotal in relieving the obstruction. During this time, the patient had an episode of nominal bowel motion once but did not pass any flatus. Conservative treatment was thus continued, and the patient was prepared for a laparoscopic diverticulectomy.

**Fig. 4:**
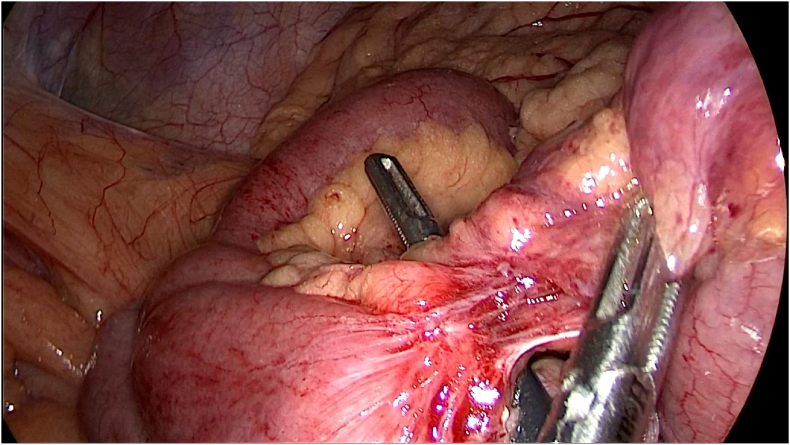
Per-operative image delineating meckel's diverticulitis with the tip attached to the ileal mesentery through the means of a band.

During the operation, meckel's diverticulum in close proximity to the ileocecal valve was confirmed. Per-operative evaluation divulged meckel's diverticulitis with the tip attached to the ileal mesentery through the means of a band (Fig. 4).

**Fig. 5:**
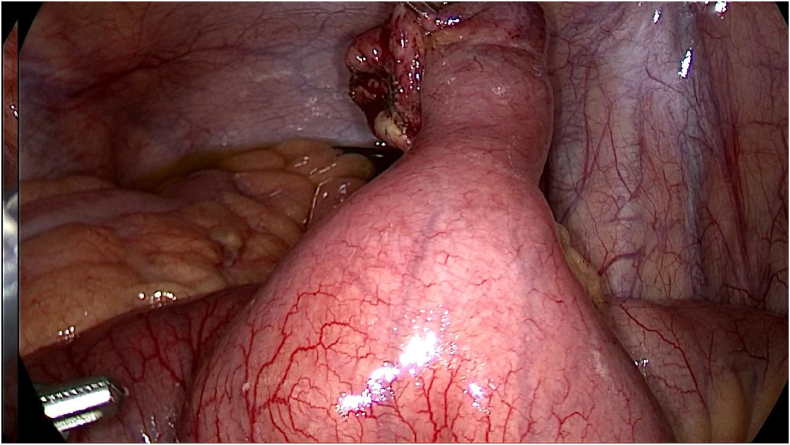
Intraoperative image demonstrating tip and band release.

Given this intraoperative finding, tip and band release was performed (Fig. 5).

**Fig. 6:**
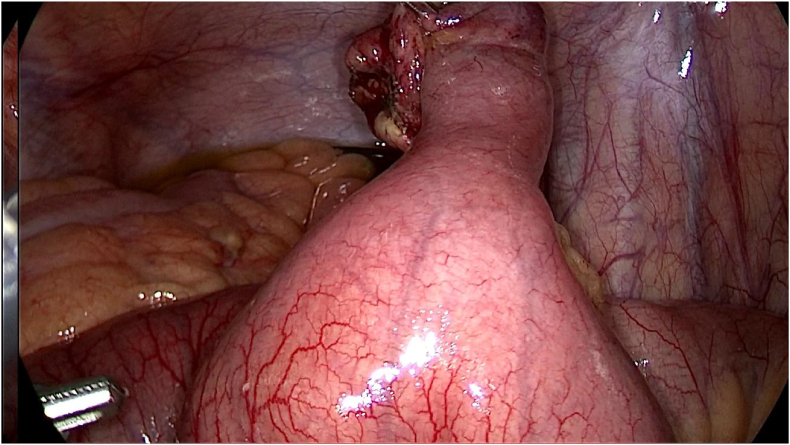
Per-operative image obtained after meckel's diverticulectomy was performed.

Additionally, given the presence of meckel's diverticulitis and the fact that the patient had been symptomatic four years prior to the current episode, meckel's diverticulectomy was performed (Fig. 6).

The surgery was conducted unremarkably, with no intra- or perioperative complications encountered during the process. Postoperatively, the patient remained well and symptom-free except for a minor wound infectious from the hypogastric port used for specimen delivery during the surgery. Daily wound dressings were advised without the need for antibiotics.

The subsequent histopathology report confirmed the presence of diffusely inflamed ectopic gastric mucosa in close proximity to the ileocecal valve, reinforcing meckel's diverticulum as the culprit etiology underlying the patient's episode.

The present paper was reported in line with the SCARE guidelines [5].

## 3 Discussion

Meckel's diverticulum (MD) is one of several congenital abnormalities that can result from incomplete obliteration of the vitelline or omphalomesenteric duct, which connects the developing intestines to the yolk sac during development, and is a true diverticulum composed of all intestinal layers [6]. It is found in roughly 2% of the population, of which only about 4% may become symptomatic due to any number of complications [6]. Specifically, small bowel obstruction (SBO) and diverticulitis secondary to ectopic gastric or pancreatic tissue are the most common presentations of symptomatic MD [7]. When it does present with obstructive symptoms, subsequent complications may arise in up to 36.5% of cases via a vast myriad of mechanisms [8]. Obstruction can commonly occur due to volvulus or torsion of the intestine around a fibrous band from MD to the umbilicus; intussusception or inversion of the MD into the ileum or cecum; or mesodiverticular band trapping the small bowel under the vascular supply of the MD and potentially leading to strangulation [9]. With symptomatic MD patients representing only 0.08% of the total population, the exceeding rarity of the condition leads to poor rates of pre-operative diagnosis, thereby obscuring the true clinical picture [9]. Early recognition is of paramount importance since a delay in surgery of 36 hours or more can triple the mortality rate from 8% to 25% in patients presenting with strangulation [10]. In this context, it is imperative that clinicians are aware of MD, its associated clinical findings, and the best practices for its diagnosis and management.

The present study elucidates a case of a 31-year-old male patient with acute onset of symptoms consistent with obstruction. The patient presented with the classical tetrad of bowel obstruction involving nausea, emesis, abdominal distension, and constipation [11]. Diagnostic imaging showed multiple air-fluid levels, which indicate pathological accumulation of fluid and gas and are a hallmark finding in X-ray and CT of SBO [12]. Ultrasound for Meckel's Diverticulum is feasible and can identify mesodiverticular bands as a hyperechoic line, particularly in pediatric patients. However, CT scan was found to be more accurate in determining the cause of small bowel obstruction in adults [13]. In cases with ectopic mucosa in the diverticular outpouch, a Meckel's radionuclide scan, which injects technetium-99 m as a dye to detect gastric tissue, can be performed [14]. A previous study found the transition zone was located near the midline in 80% of patients. In our case, the transition point was identified in the terminal or distal ileum [15].

Interestingly, volvulus of the MD was not suspected as a mechanism of obstruction in our case as there was no fibrous band connecting the MD to the umbilicus. We also did not suspect intussusception of the small bowel due to the absence of inversion of the diverticular outpouching into the ileum or cecum. The subsequent histopathology report showed unremarkable intestinal tissue. The lack of ectopic tissue thus suggests diverticulitis as a cause of inflammation, and subsequent obstruction was not a likely differential. Instead, the tip of the Meckel's diverticulum with a band attached to the ileal mesentery was discovered per-operatively during the diverticulectomy. Thus, mesodiverticular band (MDB) of Meckel's diverticulum was determined to be the cause of SBO in this patient. The MDB is a remnant of the vitelline artery, which supplies Meckel's Diverticulum and provides a bridge for bowel loops to herniate and become strangulated, gangrenous, ischemic, or otherwise mechanically obstructed [16,17].

Surgery, in specific meckel's diverticulectomy, remains the mainstay of treatment in such cases. The most common forms are diverticulectomy, wedge, or segmental resection, and the rationale for which procedure to form depends largely on the integrity of the diverticular base and proximal ileum and the location of ectopic tissue if any [3]. Wedge or segmental resection is recommended for MD with SBO; however, diverticulectomy was performed in our patient with full recovery and resolution of symptoms [3]. Generally, prophylactic removal of MD found incidentally is still a controversial topic, with one systematic review of 244 cases divulging reduced postoperative consequences for uncomplicated and asymptomatic Meckel's Diverticulum left alone [13].

In order to better elucidate the etiology underlying the obstructive symptoms seen in our case, we conducted a literature search using the digital databases (PubMed/MEDLINE, CINAHL, and Web of Science) to search for relevant material and articles implicating MD as a cause of SBO. The literature search in our search was conducted using the terms(s): “small bowel obstruction” AND “meckel's diverticulum” OR “diverticulitis” OR “volvulus” OR “intussusception”. The symptomatology, imaging findings, treatment employed, and the follow-up are delineated by Table 1 below [[18], [19], [20], [21], [22], [23], [24], [25], [26], [27], [28], [29], [30], [31], [32], [33], [34], [35], [36], [37], [38], [39], [40], [41], [42], [43], [44], [45], [46], [47], [48], [49], [50], [51], [52], [53], [54], [55], [56], [57], [58], [59]].

**Table 1:** Cases implicating MD as a causative etiology underlying SBO.

Author	Year	Age	Sex	Dx Imaging	Imaging finding	Surgery	Follow up	Treatment	Symptoms
Shelat et al. [18]	2011	15	F	Abd & chest x-ray and CT of Abd and pelvis	Mild dilatation of the small bowels, particularly in the distal jejunum and proximal ileum with thickening of the bowel wall and submucosal oedema. No transition point was seen on the CT scan	Exploratory laparotomy	Alive, no complications	Segment of the terminal ileum containing the MD and adhesion band was resected and stapled anastomosis with linear staples was performed	Colicky central abdominal pain associated with loss of appetite and nausea.
Luu et al. [19]	2016	34	NA	CT of Abd and pelvis	Dilated small bowel loops & non-propulsive peristalsis and small bowel obstruction in the right lower abdominal quadrant	Ileo-ileostomy	Alive and well	Incision of the small intestine and resection of ileum	Abd pain, nausea, vomiting
Ying et al. [20]	2020	50	M	CT of Abd and pelvis	Several distended and fluid-filled small bowel loops throughout the abdomen with a transition point within the right lower quadrant suggestive of adhesions	Laparotomy	Alive and no issues on follow up in outpatient clinic	Scarred section of MD and the adjacent small bowel segment was resected, and a side-to-side hand-sewn anastomosis was proceeded	Abd pain, nausea, vomiting
Jabri et al. [21]	2012	26	M	CT of Abd and Abd x-ray	dilated loops of small bowel, with no free air under either diaphragm	Laparotomy	Alive and recovered	IV and resection of the MD with closure of the bowel was performed and contents of small bowel were drained into stomach	Abd pain, nausea, vomiting
Gunadi et al. [22]	2021	0.16	F	Abd x-ray	Small-bowel obstruction	Exploratory laparotomy	Alive and gradual recuperation	Segmental small-bowel resection with primary anastomosis	Abd distention, nausea, vomiting
Gunadi et al. [22]	2021	5	M	Abd x-ray	Small-bowel obstruction, perforated MD and an inflamed appendix	Small-bowel resection	Alive and recovered gradually	Primary anastomosis and appendectomy	Abd pain, nausea, vomiting
Gunadi et al. [22]	2021	1.41	F	Upper GI Series	Found no abnormality in the upper GI tract	Exploratory laparotomy	Alive and gradual recovery	Segmental small-bowel resection with primary anastomosis	Abd pain, nausea, vomiting
Thakor et al. [23]	2007	74	M	Supine abdominal x-ray and CT of abdomen	Dilated loops of small bowel and stricture in the terminal ileum of unknown etiology	Laparotomy	Alive and recovered	MD was divided to release the obstruction, mobilised and subsequently removed	Cardinal symptoms, abd pain
Ebrahimi et al. [24]	2021	24	M	Abd CT	Distal small bowel obstruction	Diagnostic laparoscopy	Alive and recovered	MD was exteriorized through a laparotomy and small bowel resection with a side-to-side stapled anastomosis was performed.	Crampy abdominal pain and vomiting
Ebrahimi et al. [24]	2021	56	M	Abd CT	Distal small bowel obstruction	Diagnostic laparoscopy	Alive and recovered	MD was exteriorized and tethered to the mesentery through a band containing the diverticular blood supply	Crampy abdominal pain, vomiting and obstipation
Almetaher et al. [25]	2020	3–7	M	Abd CT	Small bowel obstruction	Laparotomy	Alive and recovered	IV given and small intestinal loops proximal to the obstruction was resected together with MD and the continuity of the bowel was restored with end-to-end anastomosis	Abd pain and vomiting
Bains et al. [26]	2021	30	F	Abd X-ray and Abd CT	Dilated jejunal and proximal ileal loops	Laparoscopic procedure	Alive and good health	Small midline incision at the umbilicus and ileo-ileal anastomosis performed	GI bleeding and acute Abd pain
Benjelloun et al. [27]	2009	28	M	Supine abdominal x-rays and Abd CT	Dilated small-bowel loops with air-fluid levels and lesion in the left upper quadrant with dilated small bowel loops proximally	Laparotomy	Alive and recovered	Intussusception was milked, and localized ileal resection with MD was undertaken	Abd pain, nausea and bilious vomiting
Dutta et al. [28]	2009	55	M	Abd X-ray and CT Abd	Non-obstructive bowel pattern and complete mid to distal small bowel obstruction	Laparotomy	No follow up mentioned	MD was exteriorized	Mid-lower and sharp Abd pain
Nunes et al. [29]	2009	47	M	Ultrasound scan	Fluid filled area containing echogenic components in the right iliac fossa with a trace of free fluid surrounding it and antimesenteric diverticulum	Lower midline laparotomy	Alive and recovered well	Resection of small bowel segment	Colicky central Abd pain and diarrhoea
Zhang et al. [30]	2020	45	F	Abd CT	Focal dilatation and thickening of the small bowel loop	Exploratory laparotomy	Alive and symptom-free and has restored normal activity and diet	Adhesiolysis and resection of the MD with the gangrenous bowel with anastomosis was performed	Abd pain accompanied by nausea and vomiting
Ekwunife et al. [31]	2014	29	M	Not mentioned	Perforated Meckel's diverticulum was identified	Segmental ileal resection	Alive, healthy but has superficial surgical site infection	IV and antibiotics were given	Worsening Abd pain in the umbilicus region
Pitiakoudis et al. [32]	2009	18	M	CT enteroclysis	MD was found 50 cm proximal to the ileocecal valve	Exploratory laparoscopy	No follow up mentioned	MD was resected by tangential excision using an Endo-Gia-stapler and it was removed using an Endocath	Abd discomfort in right lower quadrant, vomiting and fresh blood in his stools
Bergland [33]	1963	73	F	Abd x-ray	Distended small intestinal loops with multi-level fluid and gas-filled segments	Diverticulectomy and anastomosis	No follow up mentioned	The enterolith was pushed back and removed from the lumen of the distal ileum and the proximal ileum was decompressed by suction	No symptoms mentioned
Field [34]	1959	52	M	Erect x-ray	Marked distention of the small bowel, absence of gas in the large bowel. Fluid levels in the small bowel	Diverticulectomy	No follow up mentioned	Fecalith manipulated proximally to MD	Cramping Abd pains
Christiansen et al.*,* [35]	1967	48	F	Abd x-ray	Small bowel obstruction with possible gallstone ileus	Diverticulectomy	No follow up mentioned	MD was exteriorized	NA
Marwah et al. [36]	2016	22	M	CECT Abd and ultrasound	X-ray of Abdomen revealed multiple air fluid levels and CECT of the abdomen also showed dilatation of small gut loops up to the ileum with distal ileal stricture	Colonoscopy and exploratory laparotomy	No follow up mentioned	IV, electrolyte replacement, and nasogastric aspiration and segmental ileal resection including the strictured segment and MD was done along with ileo-ileal anastomosis	Abd distension after meals
Tenreiro et al. [37]	2015	18	M	CT of Abd	Revealed wall thickening and air-fluid levels compatible with small bowel obstruction, without apparent mechanical cause	Laparotomy	Alive, remained asymptomatic	Performed a segmental ileal resection with primary anastomosis	Right lower quadrant pain
Capelao et al. [38]	2017	51	M	Abd x-ray and CT of Abd	Small bowel with air fluid levels and paucity of gas in the colon and abrupt stop of the small bowel without a clear cause	Laparotomy	No follow up mentioned	IV and MD was ligated	Abd distension, vomitus, and epigastric pain
Newme et al. [39]	2020	24	M	X-ray and USG Abd	Showed distended small bowel loops and to and fro movement of bowel loops	Laparotomy	No follow up mentioned	Terminal ileum was constricted and indurated; MD was untwirled and segmental resection of the necrosed terminal ileum and Meckel's diverticulum were done	Acute abd pain and vomiting
Sarkardeh and Sani [40]	2020	92	F	Abd X-ray	Small bowel with air-fluid levels and dilated bowel loops	Laparotomy	No follow up mentioned	IV and Segmental small bowel resection including the diverticulum was performed with a primary end to end anastomosis	Abd pain, vomitus, and distention
Jabri and Sherbini [41]	2012	26	M	Abd x-ray and CT of Abd	Dilated loops of small bowel, with no free air under either diaphragm and stricture in the ileum and collapse of the distal ileum and large bowel	Laparotomy	Alive, no complications	IV and during surgery the meso-diverticular band was separated from the mesentery, the ileal loop was released from the diverticulum. Resection of the Meckel's diverticulum with closure of the bowel was performed. The small bowel was then decompressed, and the content was gently milked into the stomach before being aspirated via the nasogastric tube	Abd pain, vomitus, and distention
Takura et al. [42]	2021	56	F	Abd CT	Small intestine was generally dilated, and there was a closed loop-like appearance near the end of the ileum and surrounding fatty tissue opacity. A strangulated bowel obstruction was suspected	Laparotomy	Alive, good progress	MD was resected	Abd pain and vomiting
Sumer et al. [43]	2010	17	M	Abd x-ray	Small intestine exhibited an air fluid level	Exploratory laparotomy	Alive, recovered well	Resection of the MD	Abd pain and vomiting
Yazgan et al. [44]	2016	35	M	Abd x-ray and CECT of Abd	Markedly dilated loops of the middle and distal small bowel with multiple air-fluid levels. Tubular fluid containing structure found in LQ, deemed MD. Collapsed distal ileum	Laparotomy	Alive, no complications	Segmental resection and primary end-to-end anastomosis were performed	Abd pain, vomiting and nausea. Abdomen distended.
Bouassida et al. [45]	2011	22	M	Abd x-ray	Displayed air fluid levels of the small bowel, no pneumoperitoneum. Diagnosed as an acute small bowel obstruction.	Laparotomy	Alive, no complications	Segmental small bowel resection and hand-sewn anastomosis was performed	Abd pain and vomiting. Abd was hard & tender
Ying and Yahng [46]	2020	50	M	Abd & chest x-ray CT of Abd and pelvis	Dilated stomach and multiple air-fluid levels respectively. multiple markedly distended and fluid-filled small bowel loops throughout the abdomen with a transition point within the right lower quadrant suggestive of adhesions	Laparotomy	Alive, no complications	Extensively scarred section of MD along with the adjacent small bowel segment was resected and a side-to-side hand-sewn anastomosis	Vomiting, abdominal pain and distension
Murruste et al. [47]	2014	41	M	Abd CT	Markedly dilated small-bowel loops with multiple air-fluid levels	Laparotomy	Alive, no complications	Approximately 20 cm of the small bowel with Meckel's diverticulum was resected	Crampy and intermittent abdominal pain, nausea and retention of stool and gases
Ramnath et al. [48]	2018	16	F	Erect X-ray Abd & CT Abd	Narrow lumen of terminal ileum two feet from ileo-cecal junction	Exploratory laparotomy	Alive, no complications	Release of constricting band and resection of diverticulum along with segment of ileum was done and end to end anastomosis of ileum was done.	Abd pain, vomiting and constipation
Skarpas et al. [49]	2020	63	F	Abd x-ray and CT of Abd	Small bowel obstruction	Exploratory laparotomy	Alive, no complications	MD band caused obstruction by trapping of bowel loop. After separating the band from the mesentery, the ileal loop was released from the diverticulum. Resection of the Meckel's diverticulum and closure of the bowel were done using a TA stapler.	Distended Abd, pain in the lower right abdominal quadrant, fever 37 °C
Gupta and Singh [50]	2011	32	M	Ultrasonography (USG) of the Abd, Erect Abdo x-ray	Revealed hyperperistaltic dilated small bowel loops and multiple air fluid levels situated in the central abdomen and to the left	Exploratory laparotomy	Alive, no complications	MD and adhesion were excised, and the small bowel freed and decompressed.	Abd pain, nausea, vomiting
Arslan et al. [51]	2020	63	M	Erect X-ray Abd & CT Abd	Few distended small bowel loops and multiple air-fluid levels. CT showed fluid accumulation in the intestinal loops and local dilatation, favoring an obstruction	Exploratory laparotomy	Alive, no complications	A 15 cm segmental small intestine was resected, including the MD and the inflammatory and fragile mesentery of the bowel loops. Then, double end-to-end anastomosis was performed manually.	Abd pain, nausea, vomiting
Cartanese et al. [52]	2011	42	M	CECT Abd and ultrasound	A transition point between dilated and collapsed small bowel in the right lower quadrant consistent with a high-grade small bowel obstruction was found.	Exploratory laparotomy	Alive, no complications	The diverticulum was resected using a GIA stapler, without small bowel resection	lower quadrant and suprapubic pain and several episodes of vomiting without flatus.
Zorn et al. [53]	2022	30	M	Abd & chest X-ray. CT of Abd	Showed dilated loop sof small bowel and a distal high-grade SBO with multiple dilated loops of small bowel throughout the abdomen measuring up to 3.5 cm in diameter. Mild Ascites	Exploratory laparotomy	Alive, no complications	A segmental small bowel resection with hand sewn primary anastomosis was performed.	Abd pain, vomiting and nausea
Malderen and Camilleri [54]	2018	49	F	CT of Abd	15-cm long dilated segment, diagnosed as localized ileal dilatation close to the Meckel's diverticulum	Laparotomy	No follow up mentioned	resection of the Meckel's diverticulum and appendix	Bloody stools
Kuru et al. [55]	2013	17	M	Abd x-Ray and USG	Mildly distended small bowel loops. Dilated small bowel loops with a small amount of fluid in the right lower quadrant	Exploratory laparotomy	Alive, recovered well	MD was resected along the flange of ileum that encompassed the vascular territory of inflamed and friable mesentery. A manual two-layer, end-to-end anastomosis was performed to restore the continuity of the small bowel	Abd pain, nausea, vomiting
Marascia [56]	2019	29	F	Abd x-ray and CT of Abd	Diffuse distention of small bowel loops without evidence of free gas within the peritoneum. high-grade distal SBO with transition point in the left iliac fossa and signs suggestive of ileo-ileal intussusception	Diagnostic laparotomy	Alive, no complications	A segmental resection of the distal ileum 10 cm proximal to the cecum with a side-to-side anastomosis was performed	Abd pain with associated vomiting, abdominal bloating, constipation, and anorexia
Benhamou [57]	1979	78	M	Abd x-ray	Small bowel obstruction with opacity in the right iliac fossa	Laparotomy	No follow up mentioned	Diverticulectomy	No symptoms mentioned
Hayee et al. [58]	2003	79	F	Abd x-ray	Opacity on the left side Gastro-graffin study: numerous small bowel diverticula of varying sizes and minimal passage of barium beyond the mid-jejunum	Enterotomy	No follow up mentioned	The stone was found impacted in the middle of the jejunum and was removed via a small enterotomy	No symptoms mentioned
DiGiacomo et al. [59]	1993	9	M	Abd x-ray	Local ileus, multiple dilated bowel loops	Appendectomy and diverticulectomy	No follow up mentioned	Fecalith was manipulated distally to the cecum	No symptoms mentioned

MD: Meckel's diverticulum.

SBO: Small bowel obstruction.

Abd: Abdominal.

## 4 Limitations

The present study discusses a case report and delineates a single-center experience dealing with an unusual etiology underlying acute small bowel obstruction. While the study yields important evidence surrounding this etiology and prompts the clinicians to aptly recognize this congenital aberration as a cause of acute small bowel obstruction, it is limited by its sample size. Further multi-centric cross-sectional studies evaluating the true, unadjusted incidence of MD as the causative etiology underlying acute small bowel obstruction will further yield robust data to support the presented conclusions.

## 5 Conclusion

MD is the most common congenital abnormality of the gastrointestinal tract, presenting in 1–3% of patients, of which about 4% may become symptomatic. When asymptomatic, it is discovered incidentally during surgical exploration or through diagnostic imaging. Various hemorrhagic, inflammatory, and obstructive complications can arise, leading to an array of presentations. Nevertheless, the presence of MD as the causative etiology underlying acute SBO remains a clinical enigma, with most cases erroneously attributed to post-operative adhesions and/or abdominal hernias. Early diagnosis and a high index of suspicion are imperative to deliver the most optimal treatment. Although relatively rare in adults, MD should be considered in the list of differentials in patients with intussusception leading to SBO, especially on a background history unremarkable for the most common etiologies causing SBO.

## Disclosure

None.

## Provenance and peer review

Not commissioned, externally peer-reviewed.

## Ethical approval

NA.

## Sources of funding for your research

NA.

## Author contribution

TA, AKA, DA, MU, ESA: conceived the idea, designed the study, and drafted the manuscript.

KA, MA, TA, OK, MA, EA: conducted comprehensive literature search, screened the studies for relevant content, and created the literature review table.

AS, MO, FSA, MK: revised the manuscript critically and refined the literature review table.

AB, MMA, MS, RA: drafted the discussion part of the manuscript, revised the final version of the manuscript critically based on the reviewer and editorial comments.

TA, SH, MFM, EM: Conceived the initial study idea, diagnosed the case, and gave the final approval for publication.

## Registration of research studies

Name of the registry: NA
Unique Identifying number or registration ID: NA
Hyperlink to your specific registration (must be publicly accessible and will be checked): NA

## Consent

Written informed consent was obtained from the patient for publication of this case report and accompanying images. A copy of the written consent is available for review by the Editor-in-Chief of this journal on request.

## Guarantor

Talal Almas.

RCSI University of Medicine and Health Sciences.

123 St. Stephen's Green.

Dublin 2, Ireland.

Talalalmas.almas@gmail.com.

## Declaration of competing interest

NA.
